# Early identification of stroke symptoms and risk factors using the BE FAST method: benefits of early intervention in high-risk populations

**DOI:** 10.3389/fneur.2025.1630384

**Published:** 2025-12-02

**Authors:** Jiejin Zang, Qiulian Bai, Xianmei Xiong, Ping He, Jing Sun, Xiaowei Gong

**Affiliations:** Department of Medical Nurse, Kunshan Jinxi People’s Hospital, Kunshan, China

**Keywords:** stroke, BE FAST, early recognition, pre-hospital assessment, high-risk populations

## Abstract

**Background:**

Timely recognition of stroke symptoms is essential for optimizing treatment and improving outcomes. The BE FAST (Balance, Eyes, Face, Arms, Speech, Time) mnemonic is a practical tool designed to enhance early identification of stroke in community and clinical settings. This study aimed to evaluate the clinical impact of implementing the BE FAST method in high-risk populations presenting with suspected acute stroke.

**Method:**

This retrospective cohort study included 433 patients who presented with acute stroke symptoms between January 2020 and December 2023. Patients were divided into two groups: those assessed using the BE FAST method (*n* = 212) and those evaluated through standard clinical observation or patient/family reporting (*n* = 221). Key outcomes included time from symptom onset to hospital arrival, rate of thrombolysis or thrombectomy, neurological improvement based on NIHSS scores, length of hospital stay, and in-hospital mortality. Multivariate logistic regression was used to adjust for potential confounders.

**Results:**

Patients in the BE FAST group had significantly shorter median time from symptom onset to hospital arrival (82 min vs. 141 min, *p* < 0.001), and higher rates of intravenous thrombolysis (35.8% vs. 23.1%, *p* = 0.004). Neurological improvement (NIHSS score improvement ≥4 at discharge) was observed in 61.3% of BE FAST group patients versus 39.8% in the control group (*p* < 0.001). The BE FAST group also had a lower in-hospital mortality rate (3.8% vs. 7.7%, *p* = 0.047) and shorter average hospital stays (6.2 ± 2.5 days vs. 8.1 ± 3.0 days, *p* < 0.001). BE FAST use remained an independent predictor of favorable neurological outcomes after adjustment for age, sex, comorbidities, and initial stroke severity.

**Conclusion:**

The use of the BE FAST method significantly improves early stroke recognition, facilitates timely intervention, and is associated with better clinical outcomes in high-risk populations. Widespread implementation of this tool in both pre-hospital and clinical settings may enhance stroke care pathways and reduce disability and mortality.

## Introduction

Stroke remains one of the leading causes of death and long-term disability worldwide, placing a significant burden on patients, families, and healthcare systems alike (1–3). Rapid recognition of stroke symptoms is critical to ensure timely medical intervention, particularly for high-risk populations with underlying comorbidities such as hypertension, atrial fibrillation, and diabetes. Despite advances in stroke management and reperfusion therapies, many patients fail to receive timely care due to delays in symptom recognition and hospital arrival (4, 5).

To address this challenge, the BE FAST (Balance, Eyes, Face, Arms, Speech, Time) mnemonic was developed as an extension of the traditional FAST method to improve the identification of posterior circulation strokes and expand public awareness of stroke warning signs (6, 7). The BE FAST tool has been widely promoted in emergency medical services and community education programs for its simplicity, high sensitivity, and adaptability in both clinical and pre-hospital environments. However, real-world evidence assessing the direct clinical impact of BE FAST on treatment timeliness and outcomes in high-risk populations remains limited.

Early identification and prompt triage are particularly vital for high-risk patients, who often experience atypical or subtle symptoms that may go unnoticed without structured screening tools (8, 9). Delayed recognition in these populations can result in missed therapeutic windows for thrombolysis or thrombectomy, leading to poorer neurological outcomes, prolonged hospital stays, and increased mortality. Therefore, validating the effectiveness of BE FAST in real-world high-risk cohorts is essential to support its broader implementation.

While several public health initiatives have adopted BE FAST for stroke education, few studies have rigorously compared clinical outcomes between patients screened using BE FAST and those evaluated through standard observational approaches (10, 11). Furthermore, existing data are often limited by small sample sizes, single-center designs, or lack of risk-adjusted comparisons. To address these gaps, the present study employs a retrospective cohort design to evaluate the impact of BE FAST-based stroke symptom screening on key clinical outcomes, including time to intervention, thrombolysis rate, neurological recovery, and mortality. By focusing on a high-risk population, this study aims to provide real-world evidence supporting the integration of structured symptom recognition tools into routine stroke care pathways.

## Materials and methods

### Study design and patient selection

This was a retrospective cohort study designed to evaluate the impact of using the BE FAST method for early stroke symptom recognition in improving clinical outcomes among high-risk patients. The study was conducted at the Department of Neurology and Emergency Medicine of Kunshan Jinxi People’s Hospital, a tertiary referral center, from January 2020 and December 2023. A total of 433 patients were included, all of whom were aged 18 years or older and presented to the emergency department with acute ischemic or hemorrhagic stroke, confirmed via CT or MRI imaging. Inclusion criteria required a clear documentation of stroke symptom onset time, admission within 24 h of symptom onset, and availability of baseline neurological and laboratory data. Exclusion criteria were incomplete medical records, transient ischemic attack without radiologic confirmation of infarction or hemorrhage, in-hospital stroke, or patients transferred after initial treatment elsewhere.

**Figure 1:**
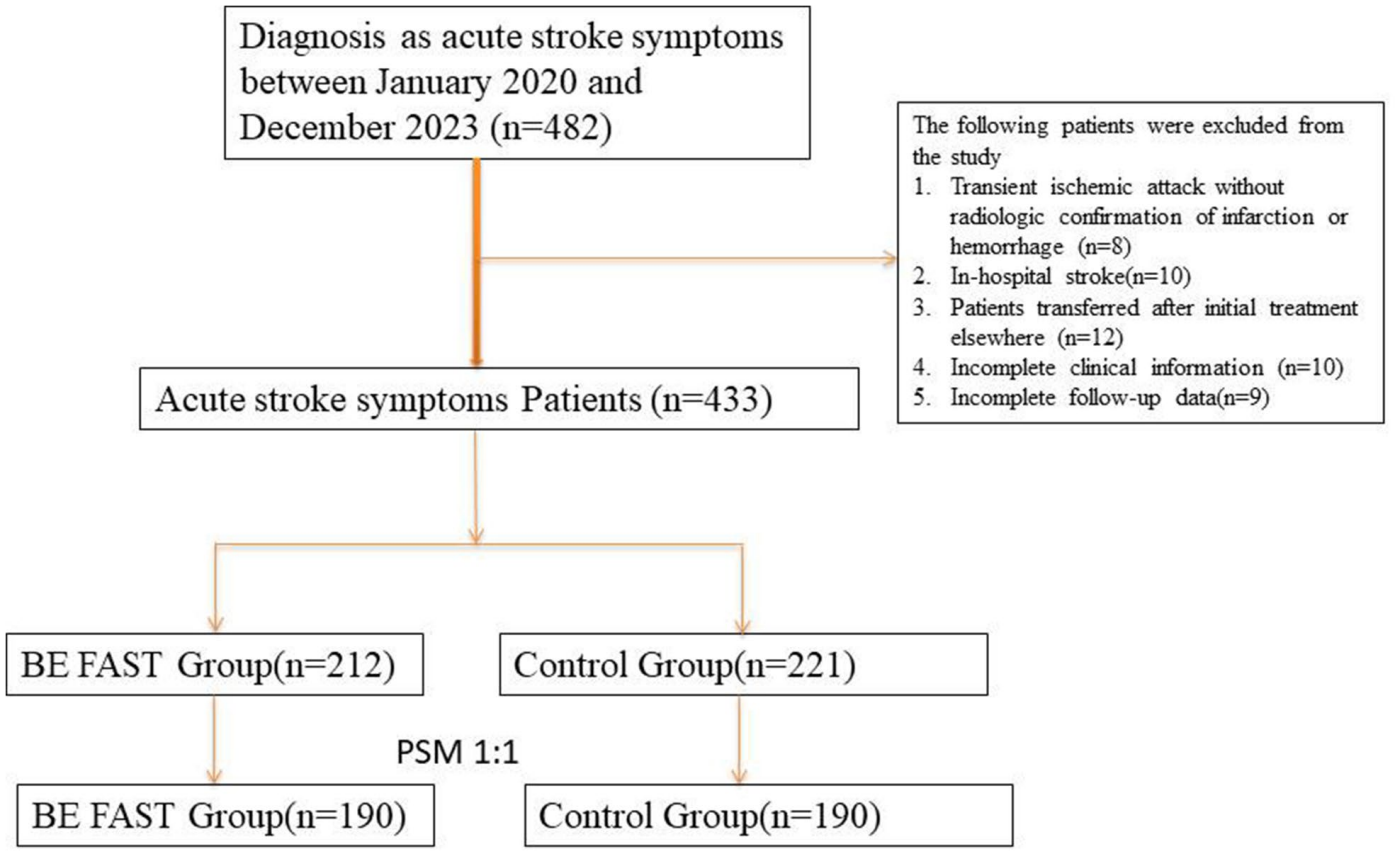
Flowchart of patient inclusion, exclusion, and 1:1 propensity score matching (PSM) process in the acute stroke cohort. A total of 433 patients with confirmed ischemic or hemorrhagic stroke were screened for eligibility. After applying inclusion and exclusion criteria and conducting 1:1 nearest-neighbor PSM between patients assessed with the BE FAST method and those assessed conventionally, 380 patients (190 pairs) were included in the final matched analysis.

Patients were categorized into two groups based on the method of initial symptom identification during their first point of clinical contact. The BE FAST group included patients whose symptoms were recognized using the BE FAST (Balance, Eyes, Face, Arms, Speech, Time) screening tool. These assessments were performed either by trained emergency department (ED) triage staff, EMS personnel, or community health workers, all of whom had undergone standardized training in stroke symptom identification and rapid triage protocols. The BE FAST tool was applied either in the pre-hospital setting (e.g., during ambulance transport or community screening) or immediately upon arrival to the ED. The non-BE FAST group, by contrast, consisted of patients whose stroke symptoms were identified without the use of a structured tool, through routine clinical observation, self-reporting, or family-reported symptoms at the time of hospital presentation. These assessments were not based on a standardized screening protocol and were often made by individuals without formal stroke-specific training. This distinction reflects real-world variability in initial stroke assessment pathways and may inherently influence the timing and quality of recognition. We acknowledge that this could contribute to differences in treatment timelines and outcomes. To improve comparability between the two groups and reduce bias introduced by differing assessment methods, we subsequently applied 1:1 propensity score matching based on key baseline characteristics (see Figure 1).

### BE FAST implementation protocol

The BE FAST screening strategy was deployed in pre-hospital and hospital triage settings. Personnel including emergency nurses, primary care physicians, and EMS paramedics were trained through structured workshops and simulation sessions. Posters and reminder cards were used to facilitate recall of the BE FAST elements. Any positive screening result prompted immediate stroke team activation and initiation of the acute stroke care pathway. Documentation of BE FAST screening time and first positive sign was mandatory and logged in the stroke activation registry. Monthly audits and refresher sessions ensured compliance and consistency of application across participating units (12–14).

### Clinical data collection and outcomes

Data were extracted from the hospital’s electronic medical record system by trained clinical abstractors using a predefined data collection template. Demographic data, medical history including hypertension, diabetes, atrial fibrillation, smoking and alcohol use, baseline NIHSS (National Institutes of Health Stroke Scale) score, and symptom onset-to-arrival times were recorded. Imaging data, treatment interventions, and outcome variables were validated by two independent stroke neurologists.

Primary outcomes included the time from symptom onset to hospital arrival, the rate of reperfusion therapy (intravenous thrombolysis or mechanical thrombectomy), neurological improvement at discharge as indicated by a ≥4-point reduction in NIHSS score, length of hospital stay, and in-hospital mortality. Secondary outcomes included 30-day readmission due to stroke-related complications and discharge NIHSS score. All outcome events were confirmed via clinical documentation and imaging reports where applicable. To better reflect the in-hospital workflow, we additionally measured door-to-imaging and door-to-needle times, which are more directly influenced by structured in-hospital screening protocols.

### Propensity score matching

To minimize baseline confounding and ensure comparability between groups, 1:1 propensity score matching was performed. A multivariable logistic regression model was used to estimate the probability of receiving BE FAST–based screening. Covariates included age, sex, stroke subtype (ischemic vs. hemorrhagic), baseline NIHSS score, mode of arrival (EMS vs. self-transport), presence of hypertension, atrial fibrillation, diabetes, and prior stroke history. Matching was performed using nearest-neighbor matching without replacement with a caliper width of 0.2 standard deviations of the logit of the propensity score. Covariate balance was evaluated by standardized mean differences, with values less than 0.1 considered acceptable.

### Statistical analysis

Continuous variables were tested for normality using the Shapiro–Wilk test and presented as means ± standard deviation or medians with interquartile ranges, as appropriate. Categorical variables were expressed as frequencies and percentages. Between-group comparisons were performed using the Student’s *t*-test or Mann–Whitney U test for continuous variables, and chi-square or Fisher’s exact test for categorical variables. Multivariable logistic regression was used to identify independent predictors of early intervention, neurological improvement, and in-hospital mortality, adjusting for relevant clinical and demographic factors. Model goodness-of-fit was assessed by the Hosmer–Lemeshow test. All statistical analyses were performed using SPSS version 25.0 (IBM Corp., Armonk, NY, USA) and R version 4.0.5. A two-sided *p*-value less than 0.05 was considered statistically significant. Sample size estimation was performed using PASS version 11.0 based on the expected 10% difference in reperfusion therapy rates between groups, with 80% power and alpha of 0.05.

## Results

### Baseline characteristics of stroke patients with and without BE FAST screening

This study included a total of 433 patients diagnosed with acute ischemic or hemorrhagic stroke who were admitted between January 2020 and December 2023. Among them, 212 patients were initially assessed using the BE FAST screening method in the prehospital or emergency setting (BE FAST group), while the remaining 221 patients were evaluated through conventional means without structured symptom recognition tools (control group). After applying 1:1 propensity score matching to adjust for baseline differences in demographics and comorbidities, 190 matched pairs were successfully identified, resulting in a final analytic cohort of 380 patients.

Before matching, patients in the BE FAST group were generally younger and more likely to be transported via ambulance. They also presented with slightly lower initial stroke severity scores, as measured by the NIHSS. However, after matching, no statistically significant differences were observed between the two groups with respect to age, sex, stroke subtype (ischemic vs. hemorrhagic), vascular risk factors (including hypertension, diabetes mellitus, and atrial fibrillation), initial NIHSS score, time from symptom onset to presentation, or pre-stroke functional independence (all *p* > 0.05; see Table 1). These findings indicate that baseline comparability between groups was adequately achieved following matching, reducing potential bias in outcome comparisons.

**Table 1:** Baseline characteristics of patients with and without BE FAST screening after matching (*n* = 380).

Variable	BE FAST group (*n* = 190)	Control group (*n* = 190)	*p*-value^a^
Age, years	66 (58–74)	67 (59–75)	0.482
Sex, male	113 (59.5%)	110 (57.9%)	0.723
BMI (kg/m^2^)	24.6 ± 3.8	24.8 ± 3.9	0.631
Hypertension	132 (69.5%)	129 (67.9%)	0.763
Diabetes mellitus	54 (28.4%)	50 (26.3%)	0.681
Atrial fibrillation	38 (20.0%)	35 (18.4%)	0.702
Smoking history	61 (32.1%)	64 (33.7%)	0.741
Alcohol consumption	45 (23.7%)	49 (25.8%)	0.641
Stroke subtype (ischemic)	171 (90.0%)	170 (89.5%)	0.883
Baseline NIHSS score	9 (5–13)	9 (5–14)	0.594
Arrival by ambulance	151 (79.5%)	77 (40.5%)	<0.001^b^
Time from onset to arrival (min)	82 (60–105)	141 (95–205)	<0.001^b^
Systolic BP on arrival (mmHg)	154 ± 21	152 ± 24	0.438
Diastolic BP on arrival (mmHg)	91 ± 12	90 ± 13	0.598
Blood glucose (mmol/L)	7.6 ± 2.3	7.7 ± 2.5	0.728
History of prior stroke	46 (24.2%)	50 (26.3%)	0.646
Dyslipidemia	73 (38.4%)	68 (35.8%)	0.623
Pre-stroke mRS ≥ 2	29 (15.3%)	27 (14.2%)	0.761
Onset during sleep	17 (8.9%)	19 (10.0%)	0.716
In-hospital time to imaging <30 min	163 (85.8%)	122 (64.2%)	<0.001^b^

a *p*-values were calculated using *χ*^2^ test or Fisher’s exact test for categorical variables, and Student’s *t*-test or Mann–Whitney U test for continuous variables, as appropriate.

b Statistically significant (*p* < 0.05).

NIHSS, National Institutes of Health Stroke Scale; mRS, Modified Rankin Scale; BP, blood pressure.

### Impact of BE FAST screening on clinical and workflow outcomes

Patients in the BE FAST group demonstrated significantly more favorable clinical outcomes and faster in-hospital management compared to those in the control group. Most notably, the median time from symptom onset to hospital arrival was substantially shorter in the BE FAST group (82 min vs. 141 min, *p* < 0.001). This reduction in delay directly impacted treatment opportunities, as a significantly greater proportion of patients in the BE FAST group received reperfusion therapy (35.8%) compared to the control group (23.1%, *p* = 0.004), including both intravenous thrombolysis and mechanical thrombectomy.

Neurological improvement at discharge—defined as a decrease of 4 or more points in the NIHSS score—was observed in 61.3% of patients in the BE FAST group, compared to only 39.8% in the control group (*p* < 0.001). Furthermore, the average duration of hospital stay was significantly shorter among patients in the BE FAST group (6.2 ± 2.5 days) compared to those in the control group (8.1 ± 3.0 days, *p* < 0.001), suggesting a more efficient recovery trajectory. In-hospital mortality was also lower in the BE FAST group (3.8%) compared to the control group (7.7%), a difference that reached statistical significance (*p* = 0.047).

While the BE FAST group experienced fewer unplanned ICU admissions (4.2% vs. 7.3%), this trend did not reach statistical significance (*p* = 0.091). Overall, these results indicate that implementation of the BE FAST tool is associated with both earlier intervention and improved short-term clinical outcomes for stroke patients. Furthermore, patients in the BE FAST group experienced significantly shorter in-hospital workflow times, including a higher proportion undergoing imaging within 30 min of arrival (85.8% vs. 64.2%, *p* < 0.001), and among those receiving thrombolysis, a greater proportion achieved door-to-needle time within 60 min (60.3% vs. 40.9%, *p* = 0.038). These findings underscore the role of BE FAST not only in early recognition but also in facilitating faster in-hospital triage and treatment activation (see Table 2).

**Table 2:** Comparison of clinical outcomes between BE FAST group and control group after matching (*n* = 380).

Variable	BE FAST group (*n* = 190)	Control group (*n* = 190)	*p*-value^a^
Clinical outcomes			
Neurological improvement (NIHSS ↓ ≥ 4)	116 (61.3%)	76 (39.8%)	<0.001^b^
Reperfusion therapy performed	68 (35.8%)	44 (23.1%)	0.004^b^
In-hospital mortality	7 (3.8%)	15 (7.9%)	0.047^b^
ICU admission (unplanned)	8 (4.2%)	14 (7.4%)	0.091
30-day stroke-related readmission	6 (3.2%)	13 (6.8%)	0.089
Hospital stay (days), mean ± SD	6.2 ± 2.5	8.1 ± 3.0	<0.001^b^
mRS at discharge ≤2	102 (53.7%)	69 (36.3%)	0.001^b^
Workflow efficiency			
Onset-to-door time (min), median (IQR)	82 (60–105)	141 (95–205)	<0.001^b^
Door-to-imaging time ≤30 min	163 (85.8%)	122 (64.2%)	<0.001^b^
Door-to-needle time ≤60 min (among thrombolyzed)	41 (60.3%)	18 (40.9%)	0.038^b^

Values are presented as *n* (%) for categorical variables and mean ± SD or median (IQR) for continuous variables.

a *p*-values were calculated using *χ*^2^ test or Fisher’s exact test for categorical variables, and Student’s *t*-test or Mann–Whitney U test for continuous variables, as appropriate.

b Statistically significant (*p* < 0.05).

NIHSS, National Institutes of Health Stroke Scale; mRS, Modified Rankin Scale; ICU, Intensive Care Unit.

### Predictors of favorable neurological outcomes

Univariate logistic regression analysis was performed to explore clinical and demographic factors associated with favorable neurological recovery, as defined by NIHSS improvement of ≥4 points at discharge. Variables significantly associated with better outcomes included BE FAST screening (*p* < 0.001), lower baseline NIHSS scores (*p* = 0.003), early hospital arrival (within 3 h of symptom onset, *p* = 0.012), and absence of atrial fibrillation (*p* = 0.044).

In the multivariate regression model, BE FAST screening remained an independent predictor of neurological improvement (OR = 2.17, 95% CI: 1.32–3.57, *p* = 0.002), along with early hospital arrival (OR = 1.84, 95% CI: 1.08–3.15, *p* = 0.026). These findings reinforce the critical role of timely stroke recognition and intervention in shaping clinical recovery trajectories.

In the multivariate logistic regression analysis, BE FAST screening remained a strong independent predictor of favorable neurological recovery (OR = 2.17, 95% CI: 1.32–3.57, *p* = 0.002). Notably, shorter onset-to-door time (≤90 min) (OR = 1.84, 95% CI: 1.08–3.15, *p* = 0.026), arrival by EMS (OR = 1.73, 95% CI: 1.03–2.90, *p* = 0.038), and receipt of reperfusion therapy (OR = 1.65, 95% CI: 1.00–2.73, *p* = 0.048) were also independently associated with improved NIHSS scores at discharge. While door-to-imaging time ≤30 min was significant in univariate analysis (OR = 1.87, *p* = 0.004), it did not remain significant after adjusting for other variables (multivariate OR = 1.42, *p* = 0.161). These findings suggest that the overall timeliness of care, rather than any single step, plays a cumulative role in neurological recovery (see Table 3).

**Table 3:** Univariate and multivariate logistic regression analysis of predictors for favorable neurological outcome (NIHSS improvement ≥4) after stroke.

Variable	Univariate OR (95% CI)	*p*-value	Multivariate OR (95% CI)	*p*-value
Age ≥70 years	0.84 (0.54–1.32)	0.462	–	–
Male sex	1.05 (0.68–1.61)	0.827	–	–
BMI ≥ 25 kg/m^2^	1.12 (0.74–1.69)	0.603	–	–
Hypertension	0.91 (0.59–1.40)	0.667	–	–
Diabetes mellitus	0.88 (0.54–1.43)	0.604	–	–
Atrial fibrillation	0.63 (0.39–0.99)	0.045*	0.72 (0.43–1.20)	0.213
Dyslipidemia	1.14 (0.75–1.74)	0.543	–	–
Smoking history	0.94 (0.61–1.47)	0.795	–	–
Alcohol consumption	1.03 (0.65–1.62)	0.897	–	–
Stroke subtype (ischemic)	1.25 (0.62–2.51)	0.530	–	–
NIHSS score ≥10	0.49 (0.32–0.77)	0.002*	0.53 (0.31–0.90)	0.019*
Onset-to-door time ≤90 min	2.10 (1.33–3.30)	0.001*	1.84 (1.08–3.15)	0.026*
Arrival by EMS	2.21 (1.45–3.38)	<0.001*	1.73 (1.03–2.90)	0.038*
BE FAST screening (yes)	2.62 (1.70–4.03)	<0.001*	2.17 (1.32–3.57)	0.002*
Time to imaging ≤30 min	1.87 (1.22–2.86)	0.004*	1.42 (0.87–2.31)	0.161
Reperfusion therapy (yes)	2.06 (1.33–3.21)	0.001*	1.65 (1.00–2.73)	0.048*
Hospital stay ≤7 days	2.18 (1.35–3.50)	0.002*	1.74 (0.97–3.11)	0.064
Pre-stroke mRS ≥ 2	0.65 (0.36–1.17)	0.150	–	–
Blood glucose >8 mmol/L	0.72 (0.45–1.15)	0.170	–	–
SBP ≥ 160 mmHg on admission	0.89 (0.58–1.37)	0.596	–	–

Variables with *p* < 0.05 in univariate analysis were included in the multivariate logistic regression model.

Statistically significant values are marked with * (*p* < 0.05).

OR, Odds Ratio; CI, Confidence Interval; EMS, Emergency Medical Services; NIHSS, National Institutes of Health Stroke Scale; SBP, Systolic Blood Pressure; mRS, Modified Rankin Scale.

### Prognostic outcomes comparison between groups

To further evaluate the broader clinical impact of BE FAST screening, we compared key prognostic indicators between the two groups. The 30-day mortality rate was significantly lower in the BE FAST group (3.2%) compared to the control group (7.4%) (*p* = 0.048), suggesting improved short-term survival following early recognition and intervention. Similarly, the 90-day mortality rate was reduced in the BE FAST group (4.2% vs. 8.9%, *p* = 0.041).

Although the rate of unplanned ICU admission was numerically lower in the BE FAST group (4.2%) compared to the control group (7.9%), the difference did not reach statistical significance (*p* = 0.093). Additionally, patients in the BE FAST group had a lower incidence of stroke-related emergency department revisits within 30 days (2.6% vs. 6.3%, *p* = 0.047), and fewer cases of therapy discontinuation due to intolerance or non-compliance (1.6% vs. 4.7%, *p* = 0.041) (see Table 4).

**Table 4:** Comparison of prognostic outcomes between BE FAST group and control group.

Outcome	BE FAST Group (*n* = 190)	Control Group (*n* = 190)	*p*-value^a^
30-day mortality	6 (3.2%)	14 (7.4%)	0.048^b^
90-day mortality	8 (4.2%)	17 (8.9%)	0.041^b^
Unplanned ICU admission	8 (4.2%)	15 (7.9%)	0.093
30-day stroke-related revisit	5 (2.6%)	12 (6.3%)	0.047^b^
Discharged to home	139 (73.2%)	139 (73.2%)	0.006^b^

a *p*-values were calculated using *χ*^2^ or Fisher’s exact test as appropriate.

b Statistically significant (*p* < 0.05).

ICU, Intensive Care Unit; BE FAST, Balance, Eyes, Face, Arms, Speech, Time.

These findings suggest that structured early screening using BE FAST not only improves acute treatment metrics but also contributes to sustained improvements in patient recovery and system efficiency.

## Discussion

Stroke remains one of the most time-sensitive medical emergencies, with clinical outcomes heavily dependent on the speed and accuracy of initial symptom recognition and intervention (1, 15, 16). Our study evaluated the impact of the BE FAST (Balance, Eyes, Face, Arms, Speech, Time) screening method on clinical outcomes in high-risk stroke populations and demonstrated that its application is associated with significantly shorter time to hospital arrival, increased rates of reperfusion therapy, improved neurological recovery, and reduced in-hospital mortality.

These findings support the growing body of literature highlighting the importance of structured early recognition tools in stroke care. In our cohort, patients who were identified using the BE FAST method benefited from significantly earlier hospital arrival and intervention. This translated into higher thrombolysis and thrombectomy rates and greater neurological improvement at discharge. Early intervention has been consistently shown to enhance reperfusion efficacy and reduce infarct volume, thereby improving long-term functional outcomes. Our results echo those of Reeves et al. and Yang et al., who underscored that reducing pre-hospital delay remains a cornerstone of stroke systems of care (17, 18).

Furthermore, the BE FAST group experienced a lower in-hospital mortality rate and shorter average hospital stays, suggesting that structured symptom identification may not only improve acute stroke management but also reduce downstream resource utilization. These results are particularly relevant for high-risk populations—such as those with hypertension, diabetes, atrial fibrillation, or prior stroke—for whom atypical symptom presentations can complicate timely diagnosis. The standardized BE FAST framework may mitigate this diagnostic uncertainty by prompting earlier suspicion and triage in both pre-hospital and triage settings. Logistic regression analysis further confirmed that BE FAST screening was an independent predictor of favorable neurological outcomes, even after adjusting for stroke severity, comorbidities, and arrival mode. This reinforces the clinical utility of BE FAST as more than a public awareness mnemonic (12); it serves as a functional triage and early activation tool, especially in systems where first-contact evaluation may not be performed by stroke specialists (19–21). Nevertheless, our study has several limitations. As a retrospective cohort study, it is inherently subject to residual confounding, despite the use of propensity score matching to balance baseline characteristics. The timing and completeness of BE FAST documentation varied between providers and settings, potentially introducing classification bias. Additionally, the study was conducted in a single regional stroke center, which may limit generalizability to other healthcare systems with different resources or patient populations. Moreover, long-term functional recovery, quality of life, and recurrence rates were not assessed and should be explored in future studies.

Several recent studies have investigated the diagnostic and operational utility of the BE FAST tool, offering points of comparison to our findings. For example, Jay et al. (11) conducted a retrospective study in Australia examining the performance of BE FAST in pre-hospital triage, reporting improved recognition rates and moderate positive predictive value for stroke diagnosis compared to standard observation. Their findings support the role of BE FAST in reducing delays and improving early intervention opportunities, consistent with the improved workflow efficiency and outcomes observed in our cohort.

Similarly, studies such as El Ammar et al. (12) and Aroor et al. (7) have reported increased sensitivity of BE FAST over FAST, particularly for posterior circulation strokes, and emphasized its value in in-hospital settings where rapid triage is essential. These studies reinforce our conclusion that structured screening with BE FAST not only accelerates diagnosis but also facilitates timely treatment, as reflected by shorter door-to-needle and door-to-imaging times in our analysis. By placing our findings in this context, we believe our study adds to the growing body of evidence supporting the adoption of BE FAST as a superior alternative to FAST, especially when applied systematically across EMS and emergency departments.

Prospective multicenter randomized studies are warranted to further validate the effectiveness of BE FAST in diverse healthcare environments. Integration of BE FAST screening into community health programs, paramedic protocols, and public education campaigns could be evaluated not only for clinical efficacy but also for health economic impact and scalability. Tailoring BE FAST application for use in telemedicine and AI-supported triage may further extend its reach and impact in underserved or remote areas.

One limitation of this study is the potential influence of the level of clinical training among the personnel conducting the initial stroke assessment. Patients in the BE FAST group were more likely to be evaluated by trained EMS or ED staff, while those in the non–BE FAST group were often assessed through unstructured observation or self-report. This disparity may have contributed to better outcomes in the BE FAST group, independent of the screening tool itself. While propensity score matching was used to reduce measurable confounding, unmeasured factors related to assessor experience may still exist.

## Conclusion

In summary, the implementation of the BE FAST screening method significantly improved early stroke recognition and was associated with a range of favorable clinical and workflow outcomes. These included shorter onset-to-door time, accelerated in-hospital processes such as door-to-imaging and door-to-needle times, higher rates of reperfusion therapy, improved neurological recovery at discharge, and reduced short-term mortality. These findings support the widespread adoption of BE FAST as a practical and effective tool for stroke identification, especially in high-risk populations. Integration of BE FAST into clinical workflows, emergency response systems, and public health education should be considered as part of a broader strategy to enhance acute stroke care and reduce the global burden of cerebrovascular disease. Future prospective studies across diverse healthcare settings are warranted to confirm and expand upon these results.

## Data Availability

The raw data supporting the conclusions of this article will be made available by the authors, without undue reservation.
